# Sugarcane smut fungus hijacks the host meristem: phytohormone-mediated sorus morphogenesis and metabolic reprogramming

**DOI:** 10.3389/fmicb.2026.1847172

**Published:** 2026-06-12

**Authors:** Shaofeng Tan, Mi Liang, Nianchen Wu, Ru Li, Lirong Chen, Chunling Zhou, Shan Lu

**Affiliations:** 1 Guangxi Sugarcane Bio-breeding Laboratory, Guangxi University, Nanning, China; 2 Guangxi Key Laboratory of Sugarcane Biology, Guangxi University, Nanning, China; 3 College of Agriculture, Guangxi University, Nanning, China; 4 State Key Laboratory for Conservation and Utilization of Subtropical Agro-Bioresources, Guangxi University, Nanning, China; 5 Ministry and Province Co-sponsored Collaborative Innovation Center for Sugarcane and Sugar Industry, Guangxi University, Nanning, China; 6 College of Life Science and Technology, Guangxi University, Nanning, China

**Keywords:** *Phytohormone homeostasis*, *Sporisorium scitamineum*, symptom development, teliospore formation, transcriptional and metabolic reprogramming

## Abstract

Disease symptom formation arises from complex interactions between a pathogen and its host. In sugarcane smut disease caused by *Sporisorium scitamineum*, the whip-shaped sorus—comprising intertwined fungal and plant tissues originating from the primary meristems of infected plants—is the most conspicuous symptom and a major cause of yield loss. Despite its agronomic significance, regulatory mechanisms underlying sorus development and teliospore formation remain poorly understood. This study investigated the dynamic molecular events underlying sorus morphogenesis using integrated phytohormone profiling, transcriptomics, and metabolomics. The data showed significant upregulation of auxin (IAA) and cytokinins (TZR, zeatin, and IPA) across developmental stages, with distinct spatial distribution patterns in the white, gray, and black sorus regions. Transcriptome analysis demonstrated extensive reprogramming of genes associated with phytohormone signaling—especially related to auxin, cytokinin, abscisic acid, and salicylic acid pathways—as well as genes involved in cell wall remodeling and transporter functions. Metabolomic profiling demonstrated accumulation of sucrose, raffinose, and hydroxycinnamic acids, and several organic acids peaked specifically prior to teliospore maturation. Fungal transcriptomic data highlighted strong upregulation of genes linked to hydrolase activity, transmembrane transport, and cell wall metabolism during teliospore differentiation. Furthermore, infection with *RWTD1* (encoding a putative four-transmembrane domain protein)-knockout mutants impaired sorus formation and triggered host defense activation without disrupting phytohormone homeostasis. Collectively, these findings provide new insights into the coordinated phytohormonal, transcriptional, and metabolic reprogramming that mediates symptom development and teliospore formation in sugarcane smut disease.

## 1 Introduction

Sugarcane (*Saccharum* spp.) is a perennial herbaceous crop in the Poaceae family with a growth cycle of 12–20 months and is cultivated predominantly in tropical and subtropical regions (Rajput et al., 2021). It is a crop of global economic importance, contributing nearly 80% of the world’s sugar supply because of the high sucrose content in its stems (Hu et al., 2024). Sugarcane is also recognized as one of the most efficient bioenergy crops for ethanol and various value-added by-products (Waclawovsky et al., 2010).

Sugarcane smut is caused by the basidiomycete *Sporisorium scitamineum* and has a complex life cycle involving nonpathogenic yeast-like haploid sporidia, infectious dikaryotic hyphae, and diploid teliospores (Yan et al., 2016). It was first reported in South Africa in 1877 (McMartin, 1945), the disease is now globally prevalent. Infected plants exhibit thinning, stunting, and excessive tillering, ultimately producing a characteristic black, whip-shaped sorus composed of fungal teliospores and plant tissue. These symptoms significantly reduce stalk yield and sugar content, especially in susceptible cultivars (Bock, 1964; Lee-Lovick, 1978). The abundant black-brown teliospores released from sori function as the primary inoculum for disease spread within or between growing seasons (Waller, 1969; Santiago et al., 2012). During suitable conditions, teliospores germinate and undergo meiosis to generate haploid basidiospores (Antoine, 1961; Alexander and Krishna, 1978). Mating between compatible sporidia produces the dikaryotic hyphae required for infection (Srinivasan, 1966). After penetrating the plant, dikaryotic hyphae proliferate within the meristem until they differentiate into diploid teliospores—a process requiring 2–12 months or more under field conditions (Antoine, 1961; Alexander and Krishna, 1978).

Interactions between sugarcane and *S. scitamineum* have been primarily investigated using transcriptomic and proteomic analyses targeting early infection stages (Barnabas et al., 2016; Schaker et al., 2016; Schaker et al., 2017; Liu et al., 2022). Previous studies show that within 24–120 h post-inoculation, smut-resistant genotypes exhibit differential expression of defense-related genes—including those involved in oxidative burst, lignification, coniferyl (CAD) and sinapyl (SAD) alcohol dehydrogenases, photorespiration, polyamine biosynthesis, flavonoid pathways, energy metabolism, and cell wall reinforcement, when compared with susceptible varieties (Que et al., 2014). Furthermore, these responses are activated earlier in resistant cultivars (Que et al., 2014). Metabolomic analyses across broader timeframes (5 and 120 days post-infection) further show that infected sugarcane plants upregulate invertases, trehalose-6-phosphate synthase, and lignin biosynthetic enzymes following sorus emergence (Schaker et al., 2016). Phytohormones are essential regulators of plant growth and development, and also play pivotal roles in plant–pathogen interactions (Wang et al., 2017). Pathogens often manipulate host phytohormone biosynthesis or signaling, or introduce their own hormone analogs, thereby driving symptom development (Wang et al., 2017). These alterations induce culm hypertrophy, abnormal vegetative growth, stunting, and formation of specialized structures such as tumors or sori (Brefort et al., 2009; Wang et al., 2017). For instance, *Ustilago maydis* infection in maize leads to substantial shifts in abscisic acid (ABA) and cytokinin (CK) profiles, including reduced CK glucosides, increased methylthiol CKs, and pronounced accumulation of *cis*-zeatin forms (Bolker et al., 2008). These hormone signatures correlate with strain virulence (Morrison et al., 2015). Similarly, infection by *Ustilago esculenta* triggers culm gall formation in *Zizania latifolia*, and this process is associated with altered levels of zeatin, zeatin riboside, and auxin (Chung and Tzeng, 2004).

The hallmark symptoms of sugarcane smut arise from two coordinated processes: formation of whip-shaped sori and differentiation of *S. scitamineum* hyphae into teliospores. Although early host responses have been extensively characterized, molecular mechanisms governing sorus morphogenesis and teliospore development are not well understood. Our preliminary study showed that deletion of the *RWTD1* (Regulation of Whip and Teliospore Development 1) gene in one mating type of *S. scitamineum* abolished sorus formation and teliospore differentiation without impairing host infection (submitted manuscript). Therefore, we conducted an integrated phytohormone, transcriptomic, and metabolomic analysis to elucidate the dynamic regulatory networks underlying sorus development. Sugarcane plantlets inoculated with wild-type and *RWTD1*-null mutant strains were used to characterize phytohormone fluctuations during sorus formation, associated signal transduction pathways, and metabolic profiling of the sorus tissue. Together, these findings provide new insights into coordinated reprogramming of hormone signaling, gene expression, and metabolism that mediates symptom expression and teliospore formation in sugarcane smut.

## 2 Materials and methods

### 2.1 Fungal strains and growth conditions

The haploid cells of the wild-type *Sporisorium scitamineum* strains (JG35 and JG36) and the *RWTD1*-null mutants (Δ35-RWTD1) were cultured in liquid YEPS medium at 28 °C on a rotary shaker at 220 rpm for 2 days, or on solid YEPS plates at 28 °C for 2 days (Deng et al., 2018).

### 2.2 Sugarcane inoculation

Haploid strains were grown in liquid YEPS medium to an OD_600_ of 1.0, washed, and resuspended in an equal volume of sterile water. Compatible strains were mixed at a 1:1 ratio, and the roots of tissue-cultured sugarcane plantlets derived from the smut-susceptible sugarcane variety XTT22 were immersed in the mixture for 3 days (Guo et al., 2024). The inoculated plantlets were subsequently transplanted into nursery substrate as previously described.

### 2.3 Microscopic observations and photomicroscopy

For microscopic examination of meristems from non-infected sugarcane and sori from infected plants, free-hand sections were prepared using a razor blade. Sections were immediately mounted in water and observed under an Olympus BX51 light microscope equipped with a DPController software-driven camera.

### 2.4 Libraries construction and sequencing

Before sampling, shoot apical meristems of sugarcane plants inoculated with the pathogen were collected at 40 days post-inoculation for sectioning, staining, and microscopic observation to confirm the presence of mycelium within the meristematic tissues. For transcriptome sequencing, each sample group consisted of three independent biological replicates. Each replicate contained 0.3 g of infected shoot apical meristem tissue derived from at least 20 plants at similar growth stages. For samples collected from the white, gray, and black portions of sori, each sample group consisted of three independent biological replicates. Each replicate contained 0.5 g of sorus tissue derived from at least five plants at similar growth stages. Total RNA was extracted from each sample using the TRIzol Reagent (Takara, China). Then, cDNA libraries were prepared with the NEBNext^®^ Ultra™ RNA Library Prep Kit (NEB, United States) according to the manufacturer’s protocol and sequenced on an Illumina HiSeq platform.

### 2.5 Sequencing data analysis

Clean reads were generated by removing adapter-contaminated reads, poly-N sequences, and low-quality reads. All downstream analyses were performed using these high-quality reads. Assembled sequences were mapped to the complete genomes of *Saccharum officinarum* hybrid XTT22 and *S. scitamineum* JG36. Differentially expressed genes (DEGs) were identified using *q*-value of < 0.005 and |log2(fold change)| > 1 as threshold parameters. GO term and KEGG pathway enrichment analyses were conducted using the GOseq R package and KOBAS software, respectively, and corrected *p*-values < 0.05 were considered statistically significant.

### 2.6 Quantitative real-time PCR assay

First, cDNA synthesis was performed using the PrimeScript™ RT Reagent Kit (Takara, Beijing, China). Primers used in this study are listed in Supplementary Table S1. Quantitative PCR was conducted using TB Green^®^ Premix Ex Taq™ II (Takara, Beijing, China) on a LightCycler^®^ 96 system (Roche, Indiana) using the following cycling parameters: 50 °C for 2 min, 95 °C for 10 min, followed by 40 cycles of 95 °C for 15 s and 60 °C for 1 min. *Actin* gene served as the internal reference for *S. scitamineum* (Zhan et al., 2023), whereas Serine/arginine repetitive matrix protein 1 (*SARMp1*) was used as reference for the sugarcane tissues (Huang et al., 2018). Relative gene expression levels were calculated using the 2^−ΔΔCt^ method based on the Ct values for the target and reference genes (Livak and Schmittgen, 2001). Each qPCR reaction included three technical replicates, and each treatment comprised three biological replicates. Statistical significance was assessed using the Student’s *t*-test.

### 2.7 Phytohormone extraction and fractionation

The white, gray, and black regions of sorus tissues, along with shoot apical meristems from healthy plantlets and plantlets inoculated with JG35 × JG36 or Δ35-RWTD1 × JG36, were collected 40 days post-inoculation. Each sample represented pooled tissue from more than 20 plantlets, with three biological replicates per condition. Samples were ground to a fine powder in liquid nitrogen and extracted in 10 mL of acetonitrile containing 8 μL of internal standards. Homogenates were incubated at 4 °C overnight. After centrifugation at 12,000 × g for 5 min, the supernatant was transferred to a new tube. The pellet was re-extracted twice with 5 mL of acetonitrile, and supernatants were combined. Subsequently, 35 mg of C18 filler was added to the pooled supernatant and vortexed for 30 s. After centrifugation at 10,000 × g for 5 min, the final supernatant was evaporated to dryness under nitrogen. Residues were reconstituted in 400 μL of methanol and stored at −20 °C until analysis.

### 2.8 HPLC-ESI-MS/MS analysis of phytohormone

Phytohormone quantification was performed using HPLC-ESI-MS/MS with multiple reaction monitoring as previously described (Pan et al., 2010). These analyses were conducted at Ruiyuan Biotechnology Co., Ltd. (Nanjing, China) according to the manufacturer’s protocol. Briefly, 2 μL of each sample was injected onto a Poroshell 120 SB-C18 reverse-phase column (2.7 μm, 150 × 2.1 mm; Agilent, United States) using an Agilent 1290 HPLC system coupled to a SCIEX QTRAP 6500+ LC-MS/MS. Chromatographic conditions included a column temperature of 30 °C and a mobile phase consisting of methanol/0.1% formic acid (A) and water/0.1% formic acid (B). The gradient program is shown in Supplementary Table S2. Mass spectrometry was conducted in positive/negative ESI switching mode with ion spray voltages of +4500/–4000 V. The parameters were as follows: curtain gas, 15 psi; nebulizer gas, 65 psi; auxiliary gas, 70 psi; and source temperature, 400 °C. Selected reaction monitoring settings for quantifying protonated/deprotonated phytohormones are listed in Supplementary Table S3. Final hormone concentrations were normalized to initial fresh tissue weight. Statistical analyses were performed using one-way analysis of variance followed by Tukey–Kramer *post-hoc* tests to correct for unequal sample sizes. Statistically significant differences were defined as *p* < 0.05.

### 2.9 Sample preparation for metabolome

For metabolome, each sample group consisted of eight independent biological replicates. Each replicate contained 0.5 g of tissue derived from either sorus tissue or healthy sugarcane shoot apical meristems from at least five plants at similar growth stages. The samples were collected from all sorus developmental stages in 5 mL tubes containing EDTA and centrifuged at 1,500 × g and 4°C for 15 min. A 100 μL aliquot of each sample was then mixed with 400 μL of cold methanol/acetonitrile (1:1, v/v) to precipitate proteins, followed by centrifugation at 14,000 × g and 4 °C for 15 min. The resulting supernatant was reconstituted in 100 μL of acetonitrile/water (1:1, v/v) for LCMS analysis. To ensure analytical stability and reproducibility, quality control (QC) samples were generated by pooling 10 μL aliquots from all samples and analyzed alongside experimental samples. QC samples were interspersed throughout the run sequence and injected after every fifth sample.

### 2.10 LC-MS/MS analysis

LC–MS/MS analyses were performed on a UHPLC system (1290 Infinity LC, Agilent Technologies) coupled to a quadrupole time-of-flight mass spectrometer (AB Sciex TripleTOF 6600) at Shanghai Applied Protein Technology Co., Ltd. Hydrophilic interaction liquid chromatography (HILIC) was conducted using a 2.1 mm × 100 mm ACQUITY UPLC BEH column (1.7 μm, Waters, Ireland) according to a previously published protocol of Bai et al. (2020). Electrospray ionization (ESI) parameters were set according to previously described (Bai et al., 2020). Raw MS data were converted to MzXML format using ProteoWizard MSConvert and processed in XCMS for peak detection using established parameters (Bai et al., 2020). Isotope and adduct annotations were assigned with Collection of Algorithms for Metabolite Profile Annotation (CAMERA). Metabolites were identified by matching accurate m/z values (mass error < 25 ppm) and MS/MS spectra against an in-house database constructed from authentic standards. Pareto-scaled principal component analysis and orthogonal partial least squares–discriminant analysis (OPLS-DA) were performed using SIMCA-P (version 14.1, Umetrics, Umeå, Sweden). Variable importance in projection (VIP) values from the OPLS-DA model were used to assess metabolite contributions to group discrimination. Metabolites with VIP > 1 were further evaluated by Student’s *t*-test and *p* < 0.05 was considered statistically significant.

## 3 Results

### 3.1 Disease progression and development of symptoms

**FIGURE 1:**
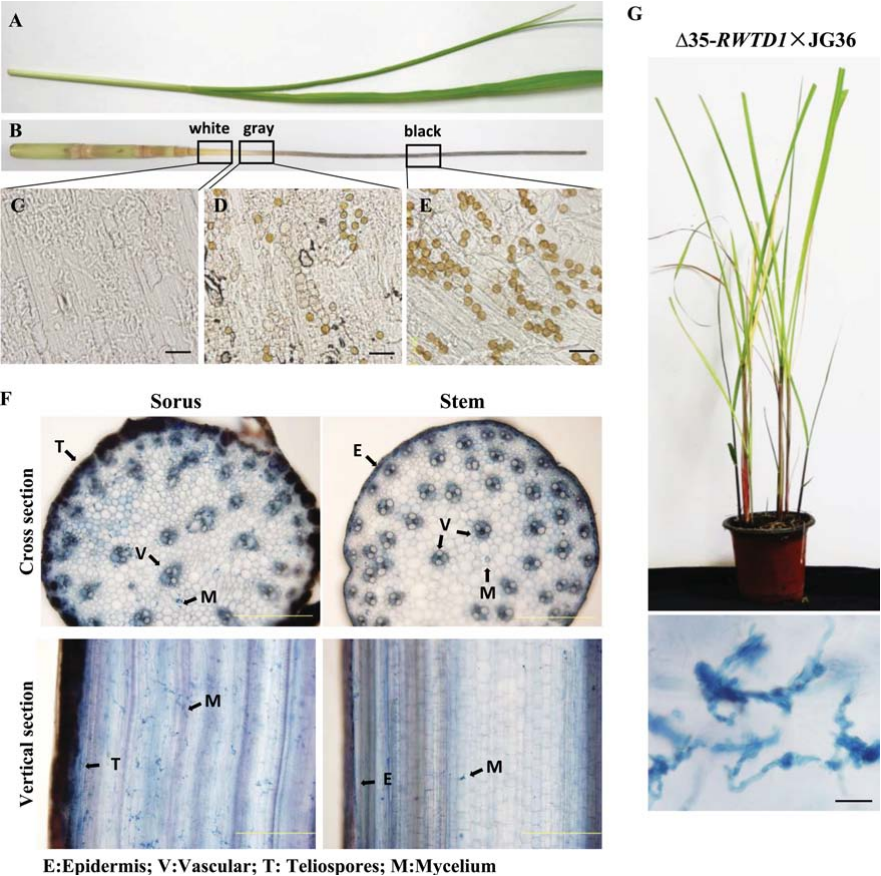
Characterization of the whip-shaped sorus and cellular morphology of *Sporisorium scitamineum* in different sorus segments of sugarcane. **(A)** Apex of a healthy sugarcane. **(B)** Whip-shaped sorus of an infected sugarcane. **(C)** Hyphal mycelium within a white sorus segment. **(D)** Mycelium and immature teliospores in a gray sorus segment. **(E)** Mature teliospores within a black sorus segment. Scale bar = 20 μm. **(F)** Cross-section of an infected sugarcane stem and sorus. Scale bar = 100 μm. **(G)** The phenotype of plantlets inoculated with Δ35-*RWTD1* × JG36 at 150 days post-inoculation and mycelium of the *RWTD1* deletion mutants within meristematic tissue. Scale bar = 20 μm.

Wild-type–infected plantlets (JG35 × JG36) exhibited severe shoot growth disorders, including transformation of the shoot apical meristem (SAM) into a whip-shaped sorus that impeded leaf differentiation (Figures 1A,B). Once initiated, the sorus elongates rapidly and emerges through the leaf sheath within 5–7 days. Structurally, the sorus resembles a modified stem and consists of plant cells, fungal hyphae, and teliospores (Figure 1F). In this structure, hyphae undergo morphological transition and produce rounded immature teliospores. The sorus is anatomically divided into three distinct regions: a basal white region, a median gray region, and an apical black region. The basal and median regions remain encased within the leaf sheath. Microscopic analysis confirmed that hyphae were abundantly present in the basal region (Figure 1C), whereas the cylindrical hyphae transitioned into transparent immature teliospores. In the median gray region, hyphae and teliospores were present, and some transparent spores matured and acquired a golden coloration (Figure 1D). The black apical region did not contain detectable hyphae and was densely packed with mature teliospores (Figure 1E). In contrast, deletion of a single *RWTD1* allele in the inoculation pair (Δ35-*RWTD1* × JG36) abolished the formation of the typical sugarcane smut “black sorus” at the plantlet apex even 150 days post-inoculation (Figure 1G). Histopathological analysis demonstrated that sorus -less mutant-infected plantlets (Δ35-*RWTD1* × JG36) were hyphae-positive (Figure 1G).

### 3.2 *S. scitamineum* reprograms endogenous phytohormone profiles in host plants to induce sorus formation

**FIGURE 2:**
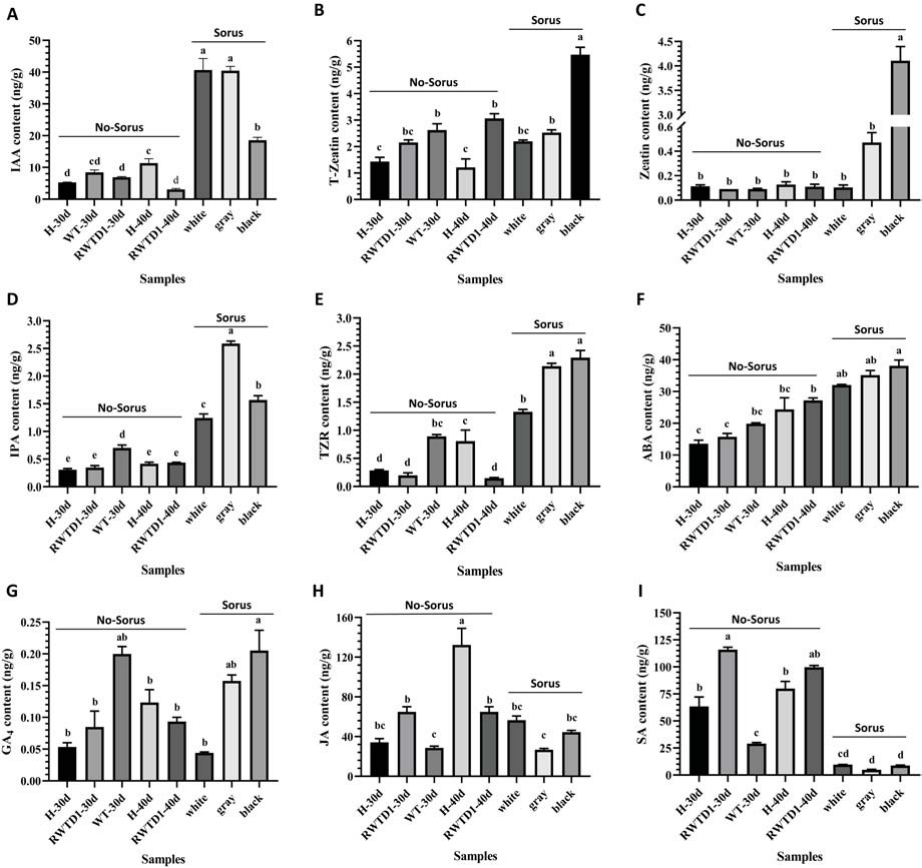
*Sporisorium scitamineum* disrupts phytohormone levels in the shoot apical meristem of sugarcane. The levels of IAA **(A)**, T-zeatin **(B)**, zeatin **(C)**, IPA **(D)**, TZR **(E)**, ABA **(F)**, GA_4_ **(G)**, JA **(H)**, and SA **(I)** in the whipless infected sugarcane meristems and three segments of the sorus. Different lowercase letters indicate statistically significant differences between groups (ANOVA with Fisher’s LSD test, *p* < 0.05).

Pathogen infection and host defense are central determinants of fungus–plant interactions. They typically elicit diverse host responses, including development of disease symptoms, formation of specialized structures, and biochemical reprogramming within infected tissues. Phytohormonal imbalance is a prominent feature of these interactions and serves as a major factor of disease-associated phenotypes, including hypertrophy, tumor formation, and differentiation of specialized structures. To characterize hormone dynamics during sorus formation, we quantified temporal changes in indole-3-acetic acid (IAA), indole propionic acid (IPA), zeatin, trans-zeatin (T-zeatin), trans-zeatin riboside (TZR), abscisic acid (ABA), gibberellic acid (GA), jasmonic acid (JA), and salicylic acid (SA) in the shoot apical meristems of healthy plantlets (H), wild-type *S. scitamineum*-infected plantlets (WT), and *RWTD1* mutant-infected plantlets (RWTD1) at 30 and 40 days post-infection (dpi), as well as in three sorus regions (white, gray, and black). Overall, the hormonal profiles were significantly different in the sorus tissues compared with the shoot apical meristems of sorus-less plantlets (Figure 2). The concentration of free IAA, the primary active auxin, was highest (∼40 ng/g) in the basal and gray sorus regions but remained below 10 ng/g in healthy and infected sorus-less shoot apical meristems (Figure 2A). In contrast, T-zeatin levels were consistently higher in the infected plantlets than in the healthy controls and reached maximum levels in the black sorus region (Figure 2B). Zeatin levels remained stable in the sorus-less meristems across time points, but increased approximately 40-fold in the black sorus region (Figure 2C). IPA levels were unchanged in the healthy and RWTD1-infected meristems but increased by ∼10-fold in the gray sorus region before declining in the black region (Figure 2D). TZR levels remained constant across all time points in the RWTD1-infected meristems, whereas its concentration at 40 dpi was 2.8-fold higher than at 30 dpi in the healthy meristems (Figure 2E). WT-infected plantlets showed significant TZR accumulation in the gray and black sorus regions (Figure 2E). ABA levels increased progressively across all sampled time points and peaked throughout the sorus structure (Figure 2F). GA4 mirrored the behavior of IPA in sorus-less samples and accumulated in the gray and black sorus regions (Figure 2G). JA remained unchanged in RWTD1-infected meristems but increased 3.8-fold in the healthy meristems at 40 dpi, with the lowest levels found in the gray sorus region (Figure 2H). In contrast with elevated levels of cytokinins in the sorus tissues, SA accumulation was significantly reduced in all sorus regions relative to sorus-less samples (Figure 2I). Collectively, these findings suggest that IAA and CTKs act synergistically to promote rapid sorus elongation and may function as signaling cues that trigger the transition of *S. scitamineum* from mycelial growth to teliospore formation.

### 3.3 Hormone and defense-related transcriptional reprogramming during sorus development and altered response to *RWTD1* mutant infection

To investigate the transcriptional regulatory networks in sugarcane during early colonization and sorus development, we sampled sorus tissues (white, gray, and black) from WT-infected plants and shoot apical meristems from healthy (H), WT-infected (WT), and *RWTD1*-infected plantlets (Δ35) at 40 dpi. RNA samples were used to generate 18 cDNA libraries and yielded 230.46 Gb of clean data. Each sample produced approximately 6.04 Gb of high-quality reads (Q30 > 94.44%; Supplementary Table S4). Alignment analyses showed that 0.49%–77.66% of reads mapped to the *S. scitamineum* genome, whereas 18.82%–90.19% mapped to the *Saccharum* hybrid XTT22 genome (Supplementary Table S4). The reliability of RNA-seq data was confirmed through RT-qPCR validation of nine genes involved in auxin-activated signaling (*ROC_gene110506*, *ROC_gene166081*, *ROC_gene206594*, *ROC_gene246729*, *ROC_gene253298*), gibberellin catabolism (*ROC_gene151979*, *ROC_gene206941*), cytokinin degradation (*ROC_gene195268*), and C-19 gibberellin 2-β-dioxygenase activity (*ROC_gene137099*; Supplementary Figure S1). Transcript abundance (Transcripts Per Kilobase of exon model per Million mapped reads, TPM) showed expression patterns consistent with the RT-qPCR results across all sample groups and confirmed the robustness of the dataset for subsequent analysis (Supplementary Figure S1).

Relative to healthy meristems, 675 DEGs were identified in WT-infected meristems prior to sorus formation. Subsequently, 26,009 DEGs were identified in the white region, 40,258 DEGs in the gray region, and 67,219 DEGs in the black region of the sorus (Supplementary Table S5). These results demonstrate extensive transcriptional reprogramming during sorus maturation, with the number of altered genes increasing sharply as sorus tissue progresses toward its fully developed state.

**FIGURE 3:**
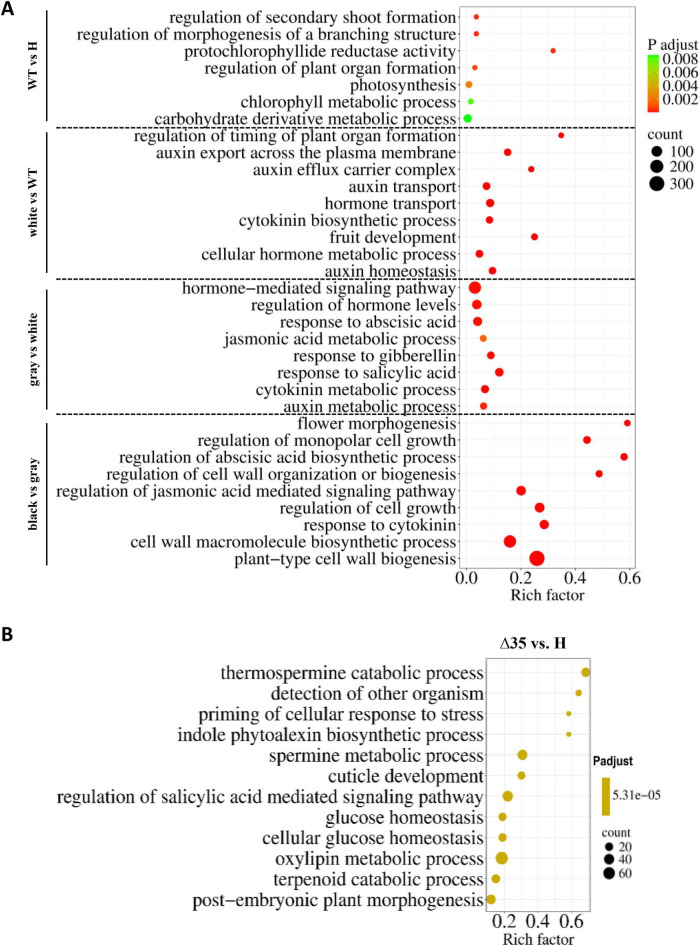
Functional enrichment analysis of the gene ontology (GO) terms for sugarcane DEGs by comparing different sorus segments and meristem tissues. **(A)** GO terms related to phytohormones and plant organ differentiation that are significantly enriched by DEGs in the WT vs. H, white vs. WT, gray vs. white, and black vs. gray datasets. Circle size indicates the number of genes. Complete information of enriched GO terms for each DEG set is provided in Supplementary Table S3. **(B)** GO term enrichment analysis of DEGs from Δ35-*RWTD1* × JG36-infected sugarcane meristem compared with the healthy sugarcane meristem.

GO enrichment analysis of the DEGs demonstrated distinct functional enrichment profiles across developmental stages. From a broad set of enriched GO terms, we focused on categories linked to organ differentiation, phytohormone regulation, and hydrolase activity. The 675 DEGs identified in the WT vs. H dataset showed significant enrichment in four GO terms—regulation of secondary shoot formation, regulation of morphogenesis of a branching structure, regulation of plant organ formation, and response to stimulus (Figure 3 and Supplementary Table S6). This suggested modified meristematic activity, which may alter apical to enable sorus initiation and promote abnormal lateral bud outgrowth. The 5,507 DEGs identified in the white vs. WT were enriched in 10 GO terms, including regulation of timing of plant organ formation, auxin export across the plasma membrane, auxin efflux carrier complex, cytokinin biosynthetic process, and auxin homeostasis (Figure 3 and Supplementary Table S6). Eight of these enriched GO terms were hormone-related. This highlighted aberrant expression of genes involved in polar auxin transport and metabolism, cytokinin biosynthesis and signaling, and several hormone regulatory pathways in the white sorus segment. The 6770 DEGs identified in the gray vs. white dataset showed enrichment in pathways linked to ABA, SA, and GA biosynthesis, metabolism, and signal transduction, including response to abscisic acid, JA metabolic process, and response to gibberellin (Figure 3 and Supplementary Table S6). Unlike the white segment, the gray segment exhibited hormone-related transcriptional changes spanning multiple hormone classes, excluding cytokinins. The transition from gray to black (31,851 DEGs) showed enrichment in GO terms associated with flower morphogenesis, regulation of monopolar and unidimensional cell growth, regulation of abscisic acid biosynthetic process, and cell wall macromolecule biosynthetic process (Figure 3 and Supplementary Table S6). Among these, four enriched GO terms were related to cell wall organization, two GO terms were related with cell growth, and one GO term was linked to floral morphogenesis. This indicated dysregulation of gene networks controlling cell expansion and organ differentiation in the black segment. Collectively, these patterns suggest that sorus development from the white to black stages likely involves hormone-mediated modulation of cell growth and differentiation, microtubule array reorganization, and apical cell expansion, which together mediate sorus elongation.

In contrast to healthy sugarcane, the 13,053 DEGs detected in meristems inoculated with the Δ35-*RWTD1* × JG36 knockout strain (Δ35 vs. H) were highly enriched in defense-related GO categories, including indole phytoalexin biosynthetic process, cellular glucose homeostasis, regulation of salicylic acid–mediated signaling pathway, and oxylipin metabolic process (Figure 3B). These enrichment patterns demonstrate that *RWTD1* knockout strains activate multiple defense-associated metabolic pathways, consistent with elevated SA levels and significantly reduce the pathogenicity of knockout strains (Figure 2I). Importantly, *RWTD1* knockout did not perturb the expression of auxin- or cytokinin-related genes in the infected meristems, nor did they induce abnormal cell proliferation.

### 3.4 Phytohormone signaling and transcriptional regulation during sorus development

**FIGURE 4:**
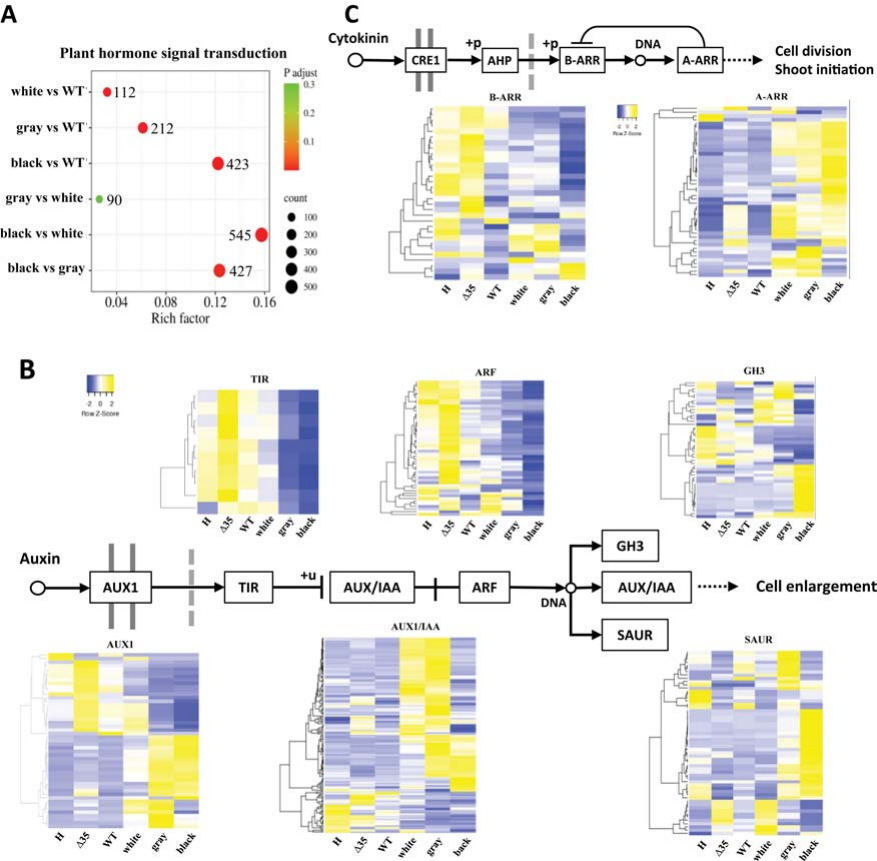
Transcriptional changes in the DEGs associated with plant hormone signal transduction. **(A)** DEGs enriched in the plant hormone signal transduction pathway across different comparative groups. Circle size indicates the number of genes. **(B)** Transcriptional trends for the DEGs in the auxin signal transduction pathway. Genes with upregulated expression are shown in yellow (z-score) and those with downregulated expression are shown in blue. **(C)** Transcriptional trends of DEGs in the cytokinin signal transduction pathway.

Comparative transcriptomic analyses across sorus development (WT vs. white/gray/black; white vs. gray/black) identified 5,507–42,742 DEGs (Supplementary Table S5). KEGG enrichment analysis revealed that 112–545 DEGs per comparison mapped to the plant hormone signal transduction pathway (Figure 4A and Supplementary Table S7). After removing redundancies, 926 unique hormone-related DEGs were identified that were related with the IAA, GA, CK, JA, ABA, ethylene (ETH), SA, and brassinosteroid signaling pathways. Among these, larger proportion of DEGs were associated with the IAA (21.2%), SA (13.7%), and ABA (13.1%) signaling pathways. These shifts in the expression of key hormone-responsive genes underscore their central roles in regulating sorus morphogenesis.

Phytohormone quantification analysis demonstrated significantly higher levels of auxin (Figure 2A) and cytokinin (Figures 2B–E) in sorus tissues and accumulation of SA and JA predominantly in the shoot apical meristems infected with the *RWTD1* knockout strains (Figures 2H,I). To determine the mechanisms by which these four hormones contribute to sorus development and how *RWTD1* deletion perturbs their signaling, we analyzed the expression profiles of genes involved in their respective transduction pathways. Auxin is a key regulator of meristem initiation and maintenance. Spatial auxin gradients are formed through polar transport and exert negative control over meristematic activity (Koshita et al., 1999). Among all the hormone pathways analyzed, the largest number of DEGs during sorus development were related with auxin signaling (Figure 4B). Furthermore, 26/49 AUX1 homologs, (53.06%) were upregulated in the gray and black sorus segments. Nine TIR1 genes and 48 ARF genes exhibited high expression prior to sorus emergence; 48 ARFs were highly expressed in non-sorus tissues but significantly downregulated in the black segment. More than half of the 54 SAUR genes were strongly expressed in the black segment. This was consistent with their roles in promoting cell elongation and organ expansion. Moreover, 39 GH3 genes were upregulated in the gray and black segments. This suggested active modulation of free IAA homeostasis to balance developmental and defense-related responses. Collectively, these data indicate that enhanced auxin biosynthesis and signaling are central factors of sorus formation (Figure 4B). Cytokinins are adenine-derived hormones that are essential for maintaining apical meristem homeostasis and positively regulating inflorescence meristem activity and floral organ initiation (D’Agostino et al., 2000). They also showed pronounced signaling changes. Forty-six cytokinin response regulators were identified in the CK pathway. Although 11 type-B ARR genes did not show any significant differential expression, 60% (21/35) of type-A ARR genes were specifically upregulated in the black segment. This suggests that enhanced expression of type-A ARRs, which function as negative feedback regulators, may suppress type-B ARR activity, thereby driving the accumulation of CKs in sugarcane (Figure 4C).

In the JA pathway, expression levels of four JAR1 and four MYC2 genes were significantly higher prior to sorus initiation compared with the white or gray segments (Supplementary Figure S2). Eleven COI1 and 76 JAZ genes were upregulated in the WT tissues and likely contributed to the suppression of JA hyperactivation through negative feedback (Supplementary Figure S2A). In SA signaling, 39 NPR1 homologs were significantly upregulated in the pre-sorus tissues whereas 15 PR1 and 55 TGA genes were strongly induced in the black segment (Supplementary Figure S2B).

### 3.5 Increased substance transport during differentiation from mycelia to teliospores in *S. scitamineum* in the sorus

To investigate the global transcriptional dynamics of *S. scitamineum* during its transition from meristematic mycelia to teliospores within developing sori, we analyzed pathogen-derived transcripts from shoot apical meristems and three sorus segments (white/SsW1, gray/SsW2, black/SsW3). Meristem samples displayed comparable pathogen biomass across treatments, and reads mapping to *S. scitamineum* accounted for 0.84% in the WT-infected plantlets, and 1.04% in the Δ35-*RWTD1* × JG36 infected plantlets. The fungal load increased sharply along the sorus axis, with mapping rates of 13.81% (SsW1), 42.41% (SsW2), and 74.85% (SsW3; Supplementary Table S4). Transcriptome profiling across segments revealed extensive stage-specific reprogramming. The data showed 387 upregulated and 578 downregulated genes in SsW1 vs. WT; 266 upregulated and 106 downregulated genes in SsW2 vs. SsW1; and 769 upregulated and 530 downregulated genes in SsW3 vs. SsW2 (Supplementary Figure S3A). RT-qPCR results validated the expression patterns of nine DEGs (Supplementary Figure S3B), thereby supporting robustness of the RNA-seq data.

**FIGURE 5:**
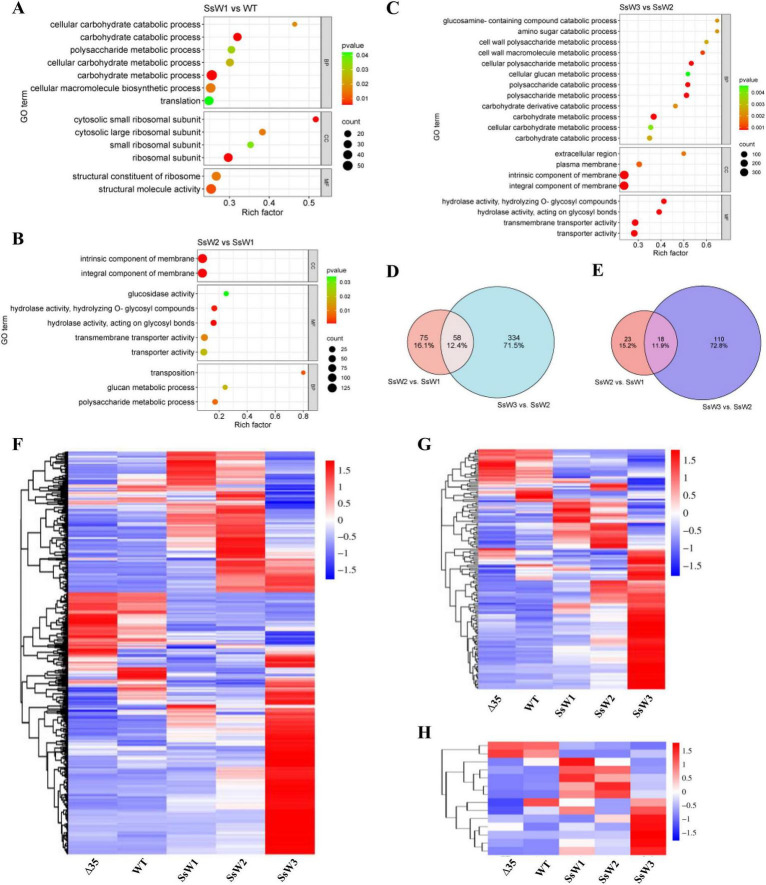
Characterization of transcriptional changes during transition of *S. scitamineum* from mycelium to teliospore in the sugarcane tissues. **(A–C)** GO enrichment analysis of DEGs from the SsW1 vs. WT **(A)**, SsW2 vs. SsW1 **(B)**, and SsW3 vs. SsW2 **(C)** comparisons datasets. BP, CC, and MF represent the biological process, cellular component, and molecular function, respectively. Circle size indicates the number of genes. Complete information of the enriched GO terms for each DEG set is provided in Supplementary Table S5. **(D,E)** Venn diagrams of membrane-related **(D)** and transport-related **(E)** DEGs from the SsW2 vs. SsW1 and SsW3 vs. SsW2 comparisons. **(F,G)** Heatmaps of *S. scitamineum* DEGs associated with membrane **(F)** and transport **(G)** functions during transition from mycelium to teliospore. **(H)** Heatmaps of DEGs enriched in the cell wall macromolecule metabolic process.

To delineate the molecular processes involved in the transition from meristematic mycelia to teliospores, we performed GO and KEGG enrichment analyses of the DEGs from each of the pairwise comparisons (SsW1 vs. WT, SsW2 vs. SsW1, SsW3 vs. SsW2). DEGs in the SsW1 vs. WT dataset were strongly enriched in carbohydrate catabolic and metabolic processes (GO:0016052, GO:0005975, GO:0044275, GO:0044262) and ribosomal subunit categories (GO:0022627, GO:0044391, GO:0022625, GO:0003735, GO:0015935) (Figure 5A and Supplementary Table S8). In SsW2 vs. SsW1 and SsW3 vs. SsW2 dataset, DEGs were enriched for integral and intrinsic membrane components (GO:0016021, GO:0031224) and transporter activity (GO:0005215), including transmembrane transport (GO:0022857; Figures 5B,C and Supplementary Table S8). Among membrane-related genes, 58 were shared between all three comparisons, 75 were unique to SsW2 vs. SsW1 and 334 were unique to SsW3 vs. SsW2 (Figure 5D). Transporter-related genes showed a similar pattern, with 18 shared between all three comparisons, 23 unique to SsW2 vs. SsW1, and 110 unique to SsW3 vs. SsW2 (Figure 5E). Cluster analyses revealed consistent expression trajectories across samples, with most membrane- and transport-associated genes weakly expressed in the meristems and ∼50% of membrane-related genes and > 50% of transporter-related genes peaking in the black segment (Figures 5F,G). Approximately 12% of transporter genes showed maximal expression in SsW1 or SsW2 (Figure 5G). In the SsW3 vs. SsW2 comparison, 14 DEGs were enriched in the cell wall macromolecule metabolic process (GO:0044036) and 12 in cell wall polysaccharide metabolism (GO:0010383) (Figure 5C). Most of these genes were suppressed in the meristem tissue (Figure 5H). Expression clustering showed that 5 genes associated with cell wall macromolecule metabolism (Figure 5H) peaked in SsW1 or SsW2, whereas 6 genes reached highest expression in SsW3 (Figure 5H).

**FIGURE 6:**
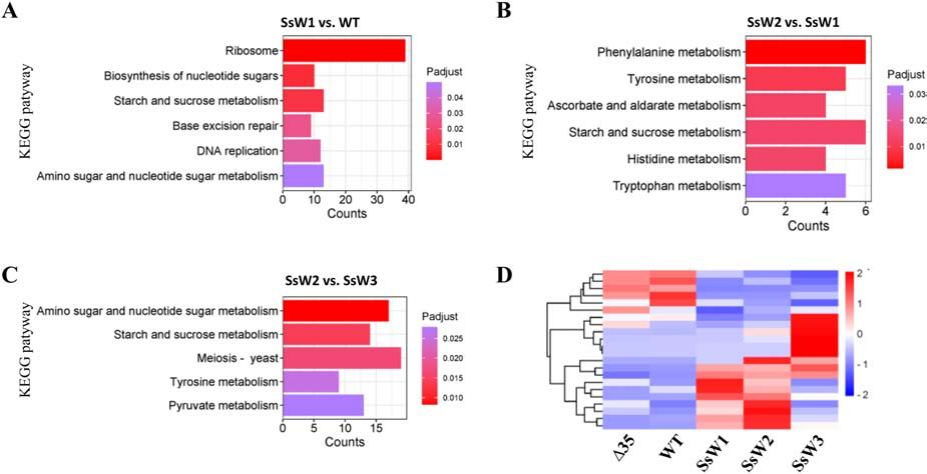
KEGG pathway enrichment analysis of *S. scitamineum* DEGs across different differentiation stages. **(A-C)** KEGG pathway enrichment analysis of DEGs from SsW1 vs. WT **(A)**, SsW2 vs. SsW1 **(B)**, and SsW3 vs. SsW2 **(C)** datasets. The X-axis indicates number of DEGs; the Y-axis shows pathway names; bar color represents adjusted *P-*value, with darker colors indicating lower *P*-values. **(D)** Heatmap of expression patterns for the DEGs associated with starch and sucrose metabolism.

KEGG enrichment analysis further highlighted stage-specific shifts in fungal metabolic pathways during sorus development. In the SsW1 vs. WT comparison, 39 DEGs associated with the ribosome (map03010) and 12 DEGs associated with DNA replication (map03030) were uniformly upregulated (Figure 6A and Supplementary Table S9), thereby indicating enhanced protein synthesis and genome duplication activity at the initial stage of *in planta* differentiation. In the SsW2 vs. SsW1 comparison, four genes enriched in histidine metabolism (map00340) were upregulated, whereas 18 of 19 meiosis-related genes (map04113) were downregulated in SsW3 vs. SsW2 (Figures 6B,C and Supplementary Table S9). This suggested repression of meiotic processes during late-stage teliospore maturation. All three comparisons showed consistent enrichment of DEGs related with starch and sucrose metabolism (map00500). Among the 22 genes examined in this pathway, 16 displayed peak expression in at least one sorus segment (Figure 6D). Collectively, these findings support a model in which *S. scitamineum* undergoes coordinated metabolic reprogramming during transition from mycelia to teliospores. This transition is characterized by increased ribosomal activity, activation of transport systems, and structural remodeling of cell membranes and walls.

### 3.6 Metabolic changes in host sugarcane caused by colonization of *S. scitamineum*

**FIGURE 7:**
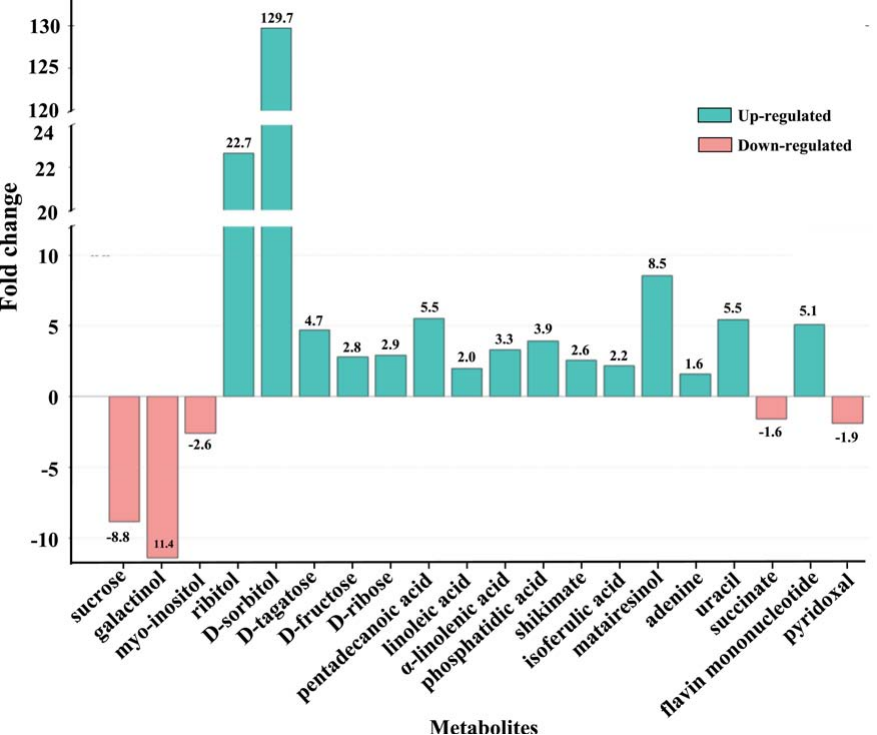
Fold change of key metabolites identified in the comparative metabolomics analysis between meristems of *S. scitamineum*-infected plantlets and healthy sugarcane plantlets. Values are presented as the fold change in metabolite abundance in the infected meristem versus healthy meristem. Green indicates upregulated metabolites; red indicates downregulated metabolites. Complete metabolite identification and statistical details are provided in Supplementary Table S10.

Infected sugarcane plants exhibit extensive metabolic alterations underlying the formation of the sorus structure, a composite of fungal hyphae and transformed host tissues. UPLC-Q-TOF/MS profiling of the three sorus segments (white, gray, and black) along with healthy meristems (H), each with eight biological replicates, revealed widespread reprogramming of primary and secondary metabolism (Figure 7 and Supplementary Table S10). Carbohydrate metabolism was highly disrupted with significant downregulation of sucrose, galactinol, and myo-inositol indicating impaired sugar turnover and energy distribution. Concurrently, significant accumulation of ribitol, D-sorbitol, D-tagatose, D-fructose, and D-ribose suggests active osmotic adjustment and potential enhancement of antioxidant capacity. Lipid metabolism also exhibited major shifts, with increased levels of pentadecanoic acid, linoleic acid, α-linolenic acid, and phosphatidic acid consistent with membrane remodeling and oxylipin-mediated defense activation. Secondary metabolic pathways were strongly induced. Elevated levels of shikimate and phenolic acids (isoferulic acid, matairesinol) suggest activation of phenylpropanoid-based defense responses. In nucleotide metabolism, increased levels of adenine and uracil suggest accelerated nucleotide turnover supporting defense-related biosynthesis, whereas reduced succinate suggests partial inhibition of TCA cycle activity. Additionally, elevated levels of flavin mononucleotide (FMN) indicate increased demand for redox cofactors, while decreased pyridoxal may influence amino acid metabolic flux. Together, these metabolomic signatures indicate a strategic redirection of host resources from growth to defense, involving osmotic protection, membrane signaling, and enhanced secondary metabolism.

### 3.7 Trends in metabolite changes during teliospore formation

**FIGURE 8:**
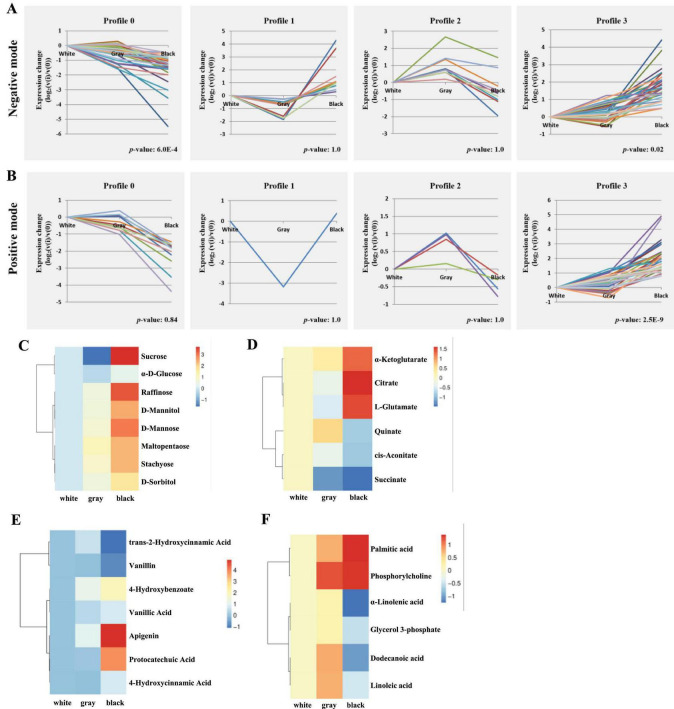
Differential metabolite profiles in different segments of the sorus structure. **(A,B)** Trend clusters of differential metabolites detected in the negative ion mode **(A)** and positive ion mode **(B)** across sorus segments. Clusters with *P*-values < 0.05 were considered statistically significant. **(C–F)** Heatmaps showing abundance patterns of differential metabolites associated with sugars and sugar alcohols **(C)**, TCA cycle intermediates **(D)**, phenylpropanoid metabolism **(E)**, and fatty acid biosynthesis-related compounds **(F)**.

Metabolic transitions underlying fungal differentiation within the sorus were further resolved by clustering metabolites based on their accumulation patterns across white, gray, and black segments. Several clusters showed significant spatial trends (Negative Profile 0, Profile 3, and Positive Profile 3; *P* < 0.05), capturing dynamic metabolic reprogramming during sorus maturation and teliospore formation (Figures 8A,B and Supplementary File 1). Sugars and secondary metabolites exhibited strong spatial regulation. The sustained accumulation of sucrose and raffinose, together with oligosaccharides (stachyose, maltopentaose) and sugar alcohols (D-sorbitol, D-mannitol), in the black segment likely supports high fungal energy demands and may modulate host sugar signaling, thereby promoting sorus elongation and teliospore development (Figure 8C). Contrary to a predicted uniform decline, organic acids displayed complex, region-specific regulation. Cis-aconitate decreased in the black segment (–0.85), whereas key TCA intermediates—citrate (1.61) and α-ketoglutarate (1.29)—accumulated significantly (Figure 8D). This pattern suggests partial interruption of the TCA cycle that redirects carbon skeletons toward biosynthetic pathways to support sporogenesis. Phenylpropanoid metabolism also showed pronounced spatial modulation. Protocatechuic acid and apigenin levels increased sharply in the black region (3.82 and 4.90, respectively), whereas 4-hydroxybenzoate levels gradually increased (1.97). Vanillic acid levels showed a modest rise (0.77), whereas trans-2-hydroxycinnamic acid and vanillin levels were substantially reduced (–1.11 and –0.80; Figure 8E). These divergent trends suggest branch-specific regulation of phenylpropanoid pathways that may reinforce fungal or host-derived structural components and enhance antioxidant protection during teliospore maturation. These metabolite patterns are consistent with transcriptomic signatures of altered cell wall remodeling, sugar transport, and phenylpropanoid biosynthesis. Fatty acid biosynthesis–related metabolites exhibited two contrasting accumulation patterns. Palmitic acid and glycerol-3-phosphate accumulated steadily, reaching 1.39 and 1.36 in the black region, indicating persistent *de novo* synthesis of saturated fatty acids and phospholipids to support extensive membrane formation required for hyphal expansion and teliospore production (Figure 8F). In contrast, dodecanoic acid, linoleic acid, and α-linolenic acid first increased in the gray region and then declined sharply in the black region (Figure 8F). This pattern indicates transient activation followed by rapid consumption of these lipids, likely through β-oxidation to meet the high energy demands of sporogenesis.

## 4 Discussion

Pathogen attack and host defense are fundamental components of plant–microbe interactions, typically activating cascades of biochemical events that culminate in disease symptoms and specialized infection structures. Smut diseases are caused by basidiomycete fungi in the Ustilaginaceae family and significantly reduce yields in major gramineous crops including corn, wheat, and sugarcane. Despite belonging to closely related taxa, smut fungi induce strikingly diverse symptoms. *Ustilago maydis* forms localized tumors on the aerial organs of maize and teosinte, whereas *Ustilago esculenta* causes SAM swelling in *Zizania latifolia*. In contrast, *Sporisorium reilianum* and *Ustilago hordei* deform inflorescences and wheat heads in maize, sorghum, and barley, respectively. *S. scitamineum* produces an elongated black sorus from the sugarcane SAM. Plant growth relies heavily on SAM activity, which orchestrates leaf and floral primordia formation through tightly regulated cell differentiation. Sugarcane smut begins with an extended asymptomatic phase during which *S. scitamineum* colonizes the SAM and coexists with host tissues (Bock, 1964; Lee-Lovick, 1978; Santiago et al., 2010). During later infection stages, the SAM is completely replaced by a characteristic whip-shaped structure, which halts leaf differentiation and severely compromises normal plant development. However, the white basal region of the sorus retains a highly mitotically active intercalary meristem that supports acropetal expansion of plant cells (Marques et al., 2017).

Plant hormones are central regulators of plant–microbe interactions and govern the development of disease symptoms (Cooper and Ashby, 1998; Ashby, 2000; Audenaert et al., 2002). During *U. maydis* infection, *Zea mays* exhibits major changes in ABA and cytokinin (CK) profiles, including pronounced reductions in cytokinin glucosides and increased accumulation of c*is*-zeatin-type CKs (cisZCKs), isopentenyladenine CKs (iPCKs), and methylthiolated CKs (2MeSCKs) (Banuett and Herskowitz, 1996; Kamper et al., 2006). These increases in CKs potentially contribute to tumor enlargement (Banuett and Herskowitz, 1996; Kamper et al., 2006). Infection with different *U. maydis* strains further produces strain-specific hormonal signatures, especially in ABA and CK levels (Morrison et al., 2015). Similarly, infection of *Z. latifolia* by *U. esculenta* increases auxin and CK production, driving culm hypertrophy and flowering suppression (Guo et al., 2015; Wang et al., 2017). Although sugarcane smut presents distinct symptoms, it shares key hormonal, including accumulation of high levels of IAA and multiple CK classes (IPA, zeatin, t-zeatin, and TZR) in the sorus segments and significant depletion of SA. These trends suggest that IAA and specific CK subclasses orchestrate segment-specific elongation of the sorus. Collectively, these findings suggest that CKs are core mediators of smut symptom development, including swelling, tumorigenesis, and sorus formation. Earlier studies confirmed that *U. maydis* sporidia and dikaryons synthesize CKs and ABA *in vitro*, whereas *S. scitamineum* can produce IAA (Bolker et al., 2008; Doehlemann et al., 2008). However, the precise origin of the elevated hormone pools in the infected tissues remains unresolved because current analytical methods cannot distinguish host-derived from pathogen-derived phytohormones. Furthermore, in future studies, functional experiments—such as exogenous hormone application, inhibitor treatments, or genetic manipulation of hormone biosynthesis or signaling pathways—will be required to establish a direct causal relationship between IAA/cytokinins and sorus development.

Sugarcane is produced through intentional hybridization of *Saccharum officinarum* and *Saccharum spontaneum* and is an economically critical crop with a large and complex genome (Chen et al., 2024). Recent genomic advances—including chromosome-level assemblies for key Chinese cultivars ZZ1 and XTT22—have generated essential resources for dissecting host–pathogen interactions (Bao et al., 2024; Chen et al., 2024). Using the XTT22 genome as a reference, transcriptomic analyses of infected meristematic tissue and white sorus segments revealed significant enrichment of DEGs in phytohormone-related GO terms and KEGG pathways, consistent with the hormonal quantification results (Figure 4A). In the auxin signaling pathway, TIR1 receptor genes and ARF transcription factors were highly expressed before sorus emergence, thereby indicating enhanced hormone perception and activation of downstream transcriptional programs (Figure 4B). This transcriptional activation likely promotes meristem cell proliferation, thereby driving sorus development. In the cytokinin pathway, type-A *ARR* genes encoding negative regulatory proteins may repress type-B ARR activity, thereby reducing transcriptional inhibition of CK-responsive genes (Figure 4C). This derepression would elevate CK signaling and stimulate meristem cell division, further underscoring the integrated roles of auxin and CKs in sorus formation. In contrast, plants infected with RWTD1 knockout strains exhibited abnormally high expression of key immune transcriptional regulators—MYC2 in the JA pathway and NPR1 in the SA pathway (Supplementary Figure S2). Their upregulation may potentiate sugarcane’s defense responses and activate immune signaling, aligning with the reduced pathogenicity observed in *RWTD1* mutant strains.

The formation of characteristic sugarcane smut symptoms is governed by two interdependent processes: the development and elongation of the specialized sorus structure and the fungal transition from hyphae to teliospores. Teliospores are the chlamydospores of smut fungi and function as the primary transmission units. They are distinguished by their dark pigmentation and ornamented walls. These adaptations confer resistance to desiccation and nutrient limitation after detachment from host tissues. Transcriptional activation of ribosome (map03010) and DNA replication (map03030) pathways during early teliospore development (SsW1) highlights the intensive protein synthesis and cellular proliferation underlying initial teliospore differentiation (Figure 5A). This is accompanied by a progressive enrichment of membrane transporter genes (GO:0022857) and carbohydrate metabolism pathways, thereby indicating increasingly dynamic nutrient acquisition strategies (Figures 5B,C). Metabolomic data support these transcriptomic trends and demonstrate gradual accumulation of ten saccharides—including raffinose and sucrose—toward teliospore maturity (Figure 8C). These sugars not only serve as energy reserves but also function as signaling molecules and stimulate the synthesis of flavonoids, isoflavones, and phenylpropanoids and promote cell wall lignification (Koch, 2004; Morkunas et al., 2005; Morkunas and Bednarski, 2008; Morkunas and Ratajczak, 2014). Concurrently, the decline in defense-related metabolites such as SA suggests active suppression of host immunity during key morphological transitions (Figure 2I). Stage-specific expression patterns of *S. scitamineum* cell wall metabolic genes (GO:0044036, GO:0010383) further reflect structural remodeling associated with teliospore formation (Figure 5B). As transporter-related DEGs become increasingly prominent in the black apical segment (SsW3), the pathogen simultaneously optimizes nutrient uptake and prepares for terminal sporulation and dispersal (Figures 5F,G). Because metabolomic measurements cannot unequivocally distinguish between plant- and fungus-derived metabolites, a host–pathogen dual-mapping strategy was used in this study. Transcriptomic alignment revealed a pronounced shift from 77.7% sugarcane-mapped reads in the basal white segment to only 20.5% in the apical black segment. This was accompanied by an increase in *S. scitamineum*–mapped reads from 14.13 to 76.32%. These results indicate that sorus formation and elongation are accompanied by the progressive conversion of host resources into pathogen biomass.

**FIGURE 9:**
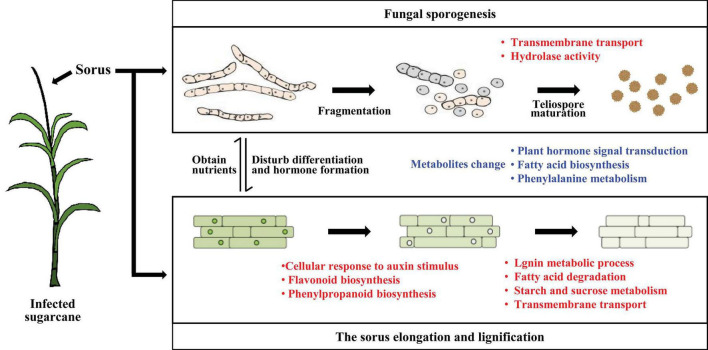
Overview of the interaction between *S. scitamineum* and sugarcane during sorus development. The proposed model for the transcriptional and metabolic reprogramming of fungal sporogenesis and sorus development. The model highlights alterations in *S. scitamineum* (upper panel) and sugarcane (lower panel) within the sorus structure. Red text indicates transcriptional changes, whereas blue text indicates metabolic changes. The fungus undergoes morphological transition from cylindrical hyphal cells to rounded, transparent immature teliospores, coinciding with the upregulated expression of genes involved in cell membrane function and transport. As teliospores mature, expression of genes related to transmembrane transport and hydrolase activity is significantly altered. Plant cells in the sorus structure gradually undergo fibrosis during upward growth. This is accompanied by transcriptional changes in genes associated with material transport, cell wall organization, fatty acid degradation, and lignin metabolism.

*S. scitamineum* orchestrates rapid sorus elongation through tightly wrapped leaf sheaths to ensure maximal teliospore exposure and enhance dispersal. The precise spatiotemporal coupling of teliospore maturation with sorus growth represents a refined parasitic strategy, likely shaped by long-term host–pathogen coevolution. Previous studies have shown that deletion of the effector gene *SsPEP1* does not prevent sorus initiation but disrupts the synchrony between sorus development and teliospore maturation and results in immature “white sorus” structures (Lu et al., 2021). Teliospore formation occurs exclusively within sorus tissues, and sporulation is not observed in non-sorus meristems. This specificity is reinforced by persistence of the hyphal stage in meristems inoculated with the Δ35-RWTD1 × JG36 mutant, where hyphae remain undifferentiated even 150 days post-inoculation (Figure 1G). Our study identifies core transcriptional and metabolic networks associated with sorus morphogenesis and teliospore development (Figure 9). However, the molecular signaling events that initiate and coordinate sorus elongation with sporulation remain unresolved.

## Data Availability

The datasets presented in this study can be found in online repositories. The names of the repository/repositories and accession number(s) can be found below: NCBI–BioProject ID PRJNA1469384.
