# Supplementary material for: Sugarcane smut fungus hijacks the host meristem: phytohormone-mediated sorus morphogenesis and metabolic reprogramming
Source: Front Microbiol. 2026 Jun 12;17:1847172. doi: 10.3389/fmicb.2026.1847172 (PMC13303569; doi:10.3389/fmicb.2026.1847172)
Supplement: Supplementary file 1 [file Table_1.docx]

**Table S1 Primers used in this study**

| **Primer name** | **Sequence (5´→3´)** |
| --- | --- |
| g_000228-qF | tcgttcttcagcctgctcatcc |
| g_000228-qR | gccgctgctgccaaacattc |
| g_000603-qF | gcgtcgttcgtgctgattacct |
| g_000603-qR | acaaagttgctgccgtcggtag |
| g_001140-qF | cgaatgctttgccgttggtgag |
| g_001140-qR | ccaaggttccagatgcgtcgtc |
| g_002901-qF | aacggctggtcacacgagagt |
| g_002901-qR | aaggtggcgagaacgctcaatg |
| g_003007-qF | gcggtacaggtggtagtggaga |
| g_003007-qR | tgagcagcagaggcatcgagat |
| g_003064-qF | cgttctccttcctccgtctcga |
| g_003064-qR | accactgtccaaccgccatct |
| g_003250-qF | tcgtctcggctcgtatgcttct |
| g_003250-qR | ttcctgctggtggtcggtca |
| g_003265-qF | gtccaagctcgttgccacctac |
| g_003265-qR | accgagtgcgaaccctgatga |
| g_003600-qF | agccaggtatgccgaccaagt |
| g_003600-qR | tgagcgtgcgaaccagtgttg |
| SARMp1-F | tggacttggtcagttggaaaca |
| SARMp1-R | tgttcctgaagcctatgttgct |
| ROC_gene110506-qF | atgtgaaggtgagcatggacgg |
| ROC_gene110506-qR | cggcagagaaggacttggtgaa |
| ROC_gene137099-qF | atctccgtgctccgctccaa |
| ROC_gene137099-qR | agacttgaggctgttggtcacc |
| ROC_gene151979-qF | ggtggtgaccaacagcctcaag |
| ROC_gene151979-qR | ttgtcgccgagcctcgtctt |
| ROC_gene166081-qF | atgtgaaggtgagcatggacgg |
| ROC_gene166081-qR | cggcagagaaggacttggtgaa |
| ROC_gene195268-qF | gcatccgtggctgaacctcttc |
| ROC_gene195268-qR | tcgggtcccacttgtgcttgt |
| ROC_gene206594-qF | tcctcctcctctgcctcccttt |
| ROC_gene206594-qR | ttctgccagtcgtcgctgct |
| ROC_gene206941-qF | ggtggtgaccaacagcctcaag |
| ROC_gene206941-qR | ttgtcgccgagcctcgtctt |
| ROC_gene246729-qF | gtcggcggctctttcgtcaaa |
| ROC_gene246729-qR | ggtctccgtccttgtcctcgta |
| ROC_gene253298-qF | tatagccacggctctccctgtc |
| ROC_gene253298-qR | tgcttgcttgctgctgtgttct |
| Actin-F | cagctcgatgaaggtcaagat |
| Actin-R | cacatctgctggaaggtagag |
