# Supplementary material for: Sugarcane smut fungus hijacks the host meristem: phytohormone-mediated sorus morphogenesis and metabolic reprogramming
Source: Front Microbiol. 2026 Jun 12;17:1847172. doi: 10.3389/fmicb.2026.1847172 (PMC13303569; doi:10.3389/fmicb.2026.1847172)
Supplement: Supplementary file 2 [file Table_2.docx]

**Table S2 Gradient parameters of HPLC**

| Time (min) | Flow rate (mL/min) | A% |
| --- | --- | --- |
| 0-1 | 0.3 | 20 |
| 1-3 | 0.3 | 20 → 50 |
| 3-9 | 0.3 | 50 → 80 |
| 9-10.5 | 0.3 | 80 |
| 10.5-10.6 | 0.3 | 80 → 20 |
