# Supplementary material for: Sugarcane smut fungus hijacks the host meristem: phytohormone-mediated sorus morphogenesis and metabolic reprogramming
Source: Front Microbiol. 2026 Jun 12;17:1847172. doi: 10.3389/fmicb.2026.1847172 (PMC13303569; doi:10.3389/fmicb.2026.1847172)
Supplement: Supplementary file 3 [file Table_3.docx]

**Table S3 Selected reaction monitoring conditions for protonated and deprotonated plant hormones ([M+H]^+^ or [M-H]^-^)**

| **Compound** | **Polarity** | **Precursor Ion** (m/z) | **Product Ion** (m/z) | **Declustering Potential** (V) | **Collision Energy** (V) |
| --- | --- | --- | --- | --- | --- |
| IAA | + | 176.1 | 130.1*/102.9 | 65 | 12/42 |
| ABA | - | 263.1 | 153.1*/204.2 | -60 | -14/-27 |
| iPR (IPA) | + | 336.2 | 136.3/204.1* | 48 | 40/45 |
| Zeatin | + | 220.3 | 136.0*/202.1 | 92 | 22/16 |
| T-Zeatin | + | 220.1 | 147.9*/202.2 | 160 | 24.5/19 |
| SA | - | 137 | 92.9*/65 | -50 | -20/-39 |
| JA | - | 209.2 | 59.1* | -54 | -16 |
| GA4 | - | 331.4 | 243.2/213.1* | -131 | -24/-39 |
| TZR | + | 352.2 | 220.1*/136/202.1 | 90 | 25/40/32 |

Note: The ion marked with an asterisk (*) is the quantifier ion.
