# Supplementary material for: Sugarcane smut fungus hijacks the host meristem: phytohormone-mediated sorus morphogenesis and metabolic reprogramming
Source: Front Microbiol. 2026 Jun 12;17:1847172. doi: 10.3389/fmicb.2026.1847172 (PMC13303569; doi:10.3389/fmicb.2026.1847172)
Supplement: Supplementary file 4 [file Table_4.docx]

**Table S4 Analysis of sequence assembly and alignment of transcriptome**

| **Sample** | **Raw reads** | **Raw bases** | **Clean reads** | **Q30 (%)** | ***S. officinarum* mapped** | ***S. scitamineum* mapped** |
| --- | --- | --- | --- | --- | --- | --- |
| H_1 | 51786448 | 7819753648 | 51192670 | 94.9 | 45893588 (89.65%) | - |
| H_2 | 50080856 | 7562209256 | 49340598 | 94.62 | 44224465 (89.63%) | - |
| H_3 | 45281988 | 6837580188 | 44703956 | 94.75 | 40194846 (89.91%) | - |
| WT_1 | 86567666 | 13071717566 | 85626868 | 94.84 | 73963505 (86.38%) | 761115 (0.89%) |
| WT_2 | 74596402 | 11264056702 | 73590702 | 94.69 | 66147595 (89.89%) | 360450 (0.49%) |
| WT_3 | 71583342 | 10809084642 | 70893202 | 95.23 | 62863772 (88.67%) | 981313 (1.38%) |
| Δ35_1 | 73237828 | 11058912028 | 72304420 | 94.91 | 64315175 (88.95%) | 780655 (1.08%) |
| Δ35_2 | 91707514 | 13847834614 | 90623530 | 95.04 | 81371352 (89.79%) | 990443 (1.09%) |
| Δ35_3 | 79773922 | 12045862222 | 78736368 | 94.83 | 70325783 (89.32%) | 811290 (1.03%) |
| White_1 | 73896538 | 11158377238 | 73060202 | 94.73 | 57605989 (78.85%) | 9543215 (13.06%) |
| White_2 | 74262210 | 11213593710 | 73521496 | 94.89 | 54862844 (74.62%) | 13395984 (18.22%) |
| White_3 | 77168204 | 11652398804 | 76309680 | 94.97 | 60836789 (79.72%) | 8478372 (11.11%) |
| Gray_1 | 82099052 | 12396956852 | 80978686 | 95.12 | 41383891 (51.1%) | 35092624 (43.34%) |
| Gray_2 | 85406574 | 12896392674 | 84430606 | 95.23 | 40134998 (47.54%) | 39780271 (47.12%) |
| Gray_3 | 70473294 | 10641467394 | 69603636 | 95.01 | 38580145 (55.43%) | 26948245 (38.72%) |
| Black_1 | 75459064 | 11394318664 | 74429770 | 94.89 | 14521913 (19.51%) | 57624949 (77.42%) |
| Black_2 | 81990376 | 12380546776 | 80901360 | 94.96 | 18402962 (22.75%) | 59780254 (73.89%) |
| Black_3 | 86420114 | 13049437214 | 85414508 | 95.07 | 16072941 (18.82%) | 66328991 (77.66%) |
