# Supplementary material for: Sugarcane smut fungus hijacks the host meristem: phytohormone-mediated sorus morphogenesis and metabolic reprogramming
Source: Front Microbiol. 2026 Jun 12;17:1847172. doi: 10.3389/fmicb.2026.1847172 (PMC13303569; doi:10.3389/fmicb.2026.1847172)
Supplement: Supplementary file 5 [file Table_5.docx]

**Table S5 Statistics of differentially expressed genes of sugarcane during whip development**

| **Group** | **Up-regulated** | **Down-regulated** | **All** |
| --- | --- | --- | --- |
| WT vs H | 400 | 275 | 675 |
| white vs H | 12029 | 13980 | 26009 |
| gray vs H | 20762 | 19496 | 40258 |
| black vs H | 28624 | 38595 | 67219 |
| ∆35 vs H | 3348 | 9705 | 13053 |
| white vs WT | 1738 | 3769 | 5507 |
| gray vs WT | 6215 | 8831 | 15046 |
| black vs WT | 12256 | 17742 | 29998 |
| white vs gray | 2212 | 4558 | 6770 |
| black vs gray | 12189 | 19662 | 31851 |
| black vs white | 19984 | 22758 | 42742 |
