# Supplementary material for: Sugarcane smut fungus hijacks the host meristem: phytohormone-mediated sorus morphogenesis and metabolic reprogramming
Source: Front Microbiol. 2026 Jun 12;17:1847172. doi: 10.3389/fmicb.2026.1847172 (PMC13303569; doi:10.3389/fmicb.2026.1847172)
Supplement: Supplementary file 10 [file Table_10.docx]

**Table S10 The differential metabolites between white vs. H**

| **Name** | **Adduct** | **Description** | **VIP** | **Fold change** | ***p*-value** | **m/z** | **rt(s)** |
| --- | --- | --- | --- | --- | --- | --- | --- |
| M360T712 | (M+NH4)+ | Sucrose | 9.38 | 0.06 | 7.46655E-12 | 360.1494835 | 712.387 |
| M298T150 | (M+H)+ | S-Methyl-5'-thioadenosine | 8.25 | 0.25 | 6.1696E-07 | 298.0965607 | 150.377 |
| M130T594 | (M+H)+ | D-Pipecolic acid | 1.78 | 0.10 | 1.5832E-06 | 130.0851445 | 594.2645 |
| M168T200 | (M+H)+ | Pyridoxal (Vitamin B6) | 2.45 | 0.43 | 7.44066E-06 | 168.0648722 | 200.139 |
| M162T796 | (M+H)+ | DL-2-Aminoadipic acid | 2.10 | 6.50 | 1.43841E-05 | 162.0750678 | 795.863 |
| M252T266 | (M+H)+ | Deoxyadenosine | 2.34 | 0.12 | 3.14455E-05 | 252.1086317 | 266.3675 |
| M198T766 | (M+NH4)+ | myo-Inositol | 1.58 | 0.38 | 4.34551E-05 | 198.0965588 | 765.972 |
| M454T448 | (M+H)+ | LysoPE(16:0/0:0) | 1.15 | 0.15 | 5.00632E-05 | 454.2911931 | 448.025 |
| M359T74 | (M+H)+ | Matairesinol | 1.48 | 8.53 | 0.000116889 | 359.1483812 | 74.2135 |
| M162T682_2 | (M+H)+ | L-Carnitine | 5.86 | 21.19 | 0.000127271 | 162.1118028 | 681.831 |
| M417T841 | (2M+H)+ | 3-(3,4-Dimethoxyphenyl)-2-propenoic acid | 1.06 | 0.35 | 0.000152003 | 417.1596089 | 840.73 |
| M204T588 | (M+H)+ | Acetylcarnitine | 1.14 | 20.00 | 0.000319592 | 204.1221633 | 588.0565 |
| M99T523 | (M+H-H2O)+ | Methylacetoacetic acid | 1.36 | 3.87 | 0.000331197 | 99.04298713 | 523.0355 |
| M146T725 | (M+H)+ | Acetylcholine | 3.97 | 33.87 | 0.000377336 | 146.1166261 | 725.1485 |
| M281T83 | (M+H)+ | Linoleic acid | 1.12 | 4.51 | 0.00059245 | 281.2464777 | 83.062 |
| M314T83 | (M+CH3CN+H)+ | 16-Hydroxypalmitic acid | 1.41 | 4.53 | 0.001027483 | 314.268918 | 83.132 |
| M184T912 | (M+H)+ | Phosphorylcholine | 4.20 | 0.53 | 0.001314342 | 184.0727381 | 912.0105 |
| M183T588_2 | (M+H)+ | D-Mannitol | 1.16 | 9.14 | 0.001409965 | 183.0851513 | 588.204 |
| M147T338 | (M+H-H2O)+ | m-Coumaric acid | 3.80 | 0.50 | 0.002218974 | 147.0431366 | 338.115 |
| M360T78 | (M+CH3COO+2H)+ | Sphingosine | 1.09 | 6.94 | 0.002364202 | 360.3103981 | 77.984 |
| M170T210 | (M+H)+ | Pyridoxine | 3.07 | 3.29 | 0.002410692 | 170.080561 | 210.0635 |
| M133T763 | (M+H)+ | L-Asparagine | 1.89 | 0.34 | 0.003055521 | 133.0597357 | 763.4555 |
| M104T623 | M+ | Choline | 2.12 | 0.59 | 0.004604102 | 104.1061433 | 623.192 |
| M258T787 | M+ | Glycerophosphocholine | 1.43 | 6.27 | 0.004903097 | 258.1094911 | 787.175 |
| M273T265 | (M+H)+ | Naringenin | 2.84 | 0.06 | 0.005079806 | 273.0753303 | 264.968 |
| M134T784_2 | (M+H)+ | L-Aspartate | 1.79 | 0.61 | 0.006613749 | 134.0439667 | 783.8235 |
| M102T84 | (M+H)+ | L-3-Aminodihydro-2(3H)-furanone | 1.02 | 0.67 | 0.008082668 | 102.0539435 | 84.248 |
| M245T305 | (M+H)+ | Uridine | 1.72 | 0.63 | 0.008191644 | 245.0760875 | 305.352 |
| M268T322 | (M+H)+ | Adenosine | 4.61 | 0.06 | 0.008803208 | 268.1033655 | 321.64 |
| M279T126 | (M+H)+ | all cis-(6,9,12)-Linolenic acid | 3.62 | 3.32 | 0.00900374 | 279.2315254 | 125.9005 |
| M265T342 | (M+H)+ | Valyl-Phenylalanine | 1.11 | 2.64 | 0.013752423 | 265.1535502 | 342.4075 |
| M123T337_2 | (M+H)+ | Erythritol | 1.65 | 43.04 | 0.015715307 | 123.0641111 | 336.915 |
| M124T424 | (M+H)+ | Nicotinate | 1.23 | 1.49 | 0.016304107 | 124.0382503 | 423.787 |
| M118T526_2 | (M+H)+ | Betaine | 4.97 | 1.78 | 0.017513877 | 118.0853099 | 526.0515 |
| M243T184 | (M+H)+ | Thymidine | 1.22 | 0.57 | 0.019836998 | 243.0970496 | 184.052 |
| M198T581 | (M+NH4)+ | D-Mannose | 1.84 | 3.70 | 0.024090242 | 198.0965539 | 581.2295 |
| M177T322 | (M+H-H2O)+ | Isoferulic acid | 1.96 | 2.17 | 0.027395188 | 177.052774 | 322.064 |
| M147T767 | (M+NH4)+ | L-Pyroglutamic acid | 1.79 | 0.34 | 0.031165031 | 147.0754397 | 766.5875 |
| M189T81 | (M+H)+ | Valyl-Alanine | 1.21 | 1.75 | 0.049356768 | 189.121989 | 80.816 |
| M341T714_2 | (M-H)- | Sucrose | 11.58 | 0.11 | 2.29416E-09 | 341.1090325 | 713.551 |
| M401T714 | (M+CH3COO)- | Galactinol | 10.11 | 0.09 | 5.12983E-09 | 401.1305188 | 713.608 |
| M128T596 | (M-H)- | L-Pipecolic acid | 1.32 | 0.10 | 1.15567E-07 | 128.0710668 | 595.6875 |
| M151T481 | (M-H)- | Ribitol | 1.91 | 22.67 | 2.78252E-07 | 151.0613264 | 480.623 |
| M267T292 | (2M-H)- | 2'-Deoxy-D-ribose | 1.36 | 0.07 | 1.03042E-06 | 267.1085911 | 291.994 |
| M166T201 | (M-H)- | Pyridoxal (Vitamin B6) | 1.18 | 0.52 | 1.2469E-06 | 166.0508519 | 201.394 |
| M181T590 | (M-H)- | D-Sorbitol | 4.94 | 129.71 | 2.56386E-06 | 181.0719698 | 589.591 |
| M179T764 | (M-H)- | myo-Inositol | 2.93 | 0.38 | 3.14549E-06 | 179.0565484 | 763.513 |
| M149T296 | (M-H)- | D-Lyxose | 1.45 | 1.57 | 2.54279E-05 | 149.0452359 | 295.962 |
| M239T515 | (M+CH3COO)- | D-Tagatose | 9.41 | 4.70 | 0.000140938 | 239.0775239 | 515.1235 |
| M134T299 | (M-H)- | Adenine | 3.65 | 1.59 | 0.000155819 | 134.0473518 | 299.321 |
| M356T153 | (M+CH3COO)- | S-Methyl-5'-thioadenosine | 2.28 | 0.20 | 0.000156342 | 356.1030903 | 153.1665 |
| M179T515 | (M-H)- | D-Fructose | 11.68 | 2.81 | 0.000168411 | 179.0565668 | 514.9795 |
| M383T508 | M- | N-Acetyllactosamine | 1.31 | 4.26 | 0.000679413 | 383.1460582 | 507.5535 |
| M241T79 | (M-H)- | Pentadecanoic Acid | 1.76 | 5.52 | 0.000686772 | 241.2174047 | 78.968 |
| M671T64 | (M-H)- | PA(16:0/18:2(9Z,12Z)) | 1.07 | 3.93 | 0.001499485 | 671.4657055 | 64.051 |
| M71T517 | (M-H2O-H)- | Dihydroxyacetone | 1.18 | 2.56 | 0.001541516 | 71.01327365 | 517.099 |
| M129T212_2 | (M-H)- | Glutaconic acid | 1.53 | 2.49 | 0.001953824 | 129.0188788 | 211.966 |
| M149T517 | (M-H)- | D-Ribose | 1.40 | 2.90 | 0.002288184 | 149.0454274 | 517.01 |
| M117T759 | (M-H)- | Succinate | 1.46 | 0.63 | 0.002363428 | 117.0190494 | 759.39 |
| M173T697 | (M-H)- | Shikimate | 2.79 | 2.56 | 0.002481656 | 173.0457559 | 697.204 |
| M283T54 | (M+Na-2H)- | D-Mannitol 1-phosphate | 1.32 | 15.17 | 0.00261356 | 283.1219138 | 54.0055 |
| M455T64 | (M-H)- | Flavin mononucleotide (FMN) | 1.67 | 5.08 | 0.002954705 | 455.1013506 | 64.2335 |
| M326T320_1 | (M+CH3COO)- | Adenosine | 1.78 | 0.07 | 0.006708583 | 326.1104963 | 319.5075 |
| M111T156 | (M-H)- | Uracil | 1.76 | 5.46 | 0.00697722 | 111.019536 | 156.163 |
| M132T780 | (M-H)- | D-Aspartic acid | 1.89 | 0.67 | 0.009129239 | 132.0303417 | 780.471 |
| M177T201 | (M-H)- | D-Glucono-1,5-lactone | 2.43 | 1.72 | 0.009989941 | 177.0407417 | 200.9895 |
| M237T199 | (M+CH3COO)- | L-Gulonic gamma-lactone | 1.98 | 2.20 | 0.012872966 | 237.0617599 | 198.576 |
| M191T668 | (M-H)- | Quinate | 5.30 | 1.74 | 0.014579913 | 191.0567552 | 668.422 |
| M279T76 | (M-H)- | Linoleic acid | 6.29 | 1.98 | 0.015263686 | 279.2333308 | 76.408 |
| M163T335 | (M-H)- | trans-2-Hydroxycinnamic acid | 1.27 | 0.60 | 0.01634646 | 163.040065 | 334.7095 |
| M277T78 | (M-H)- | alpha-Linolenic acid | 2.32 | 0.36 | 0.033934224 | 277.217211 | 77.948 |
