# Supplementary material for: Sugarcane smut fungus hijacks the host meristem: phytohormone-mediated sorus morphogenesis and metabolic reprogramming
Source: Front Microbiol. 2026 Jun 12;17:1847172. doi: 10.3389/fmicb.2026.1847172 (PMC13303569; doi:10.3389/fmicb.2026.1847172)
Supplement: Supplementary file 12 [file Data_Sheet_2.pdf]

Supplementary figures

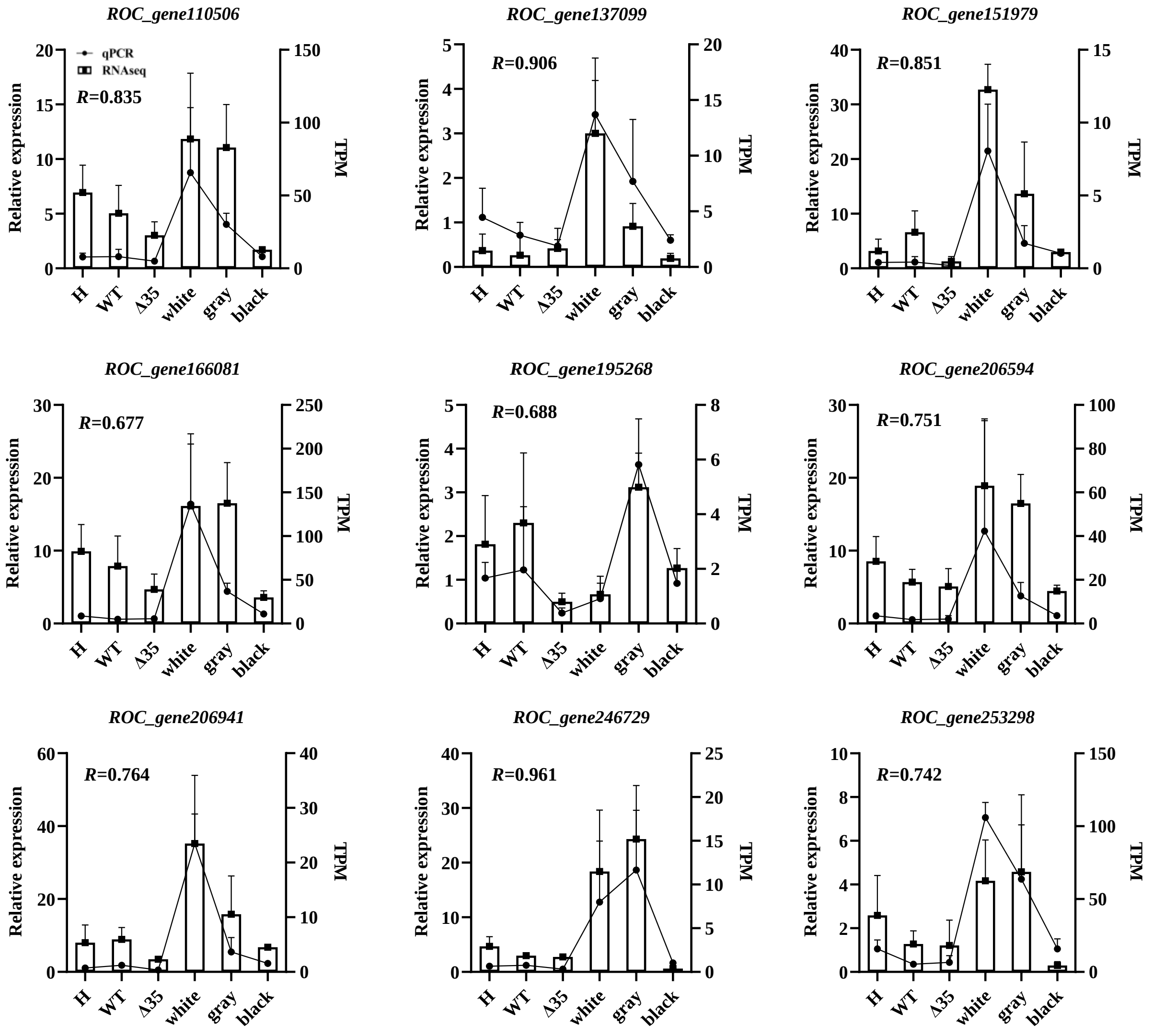

FIGURE S1 Validation of sugarcane gene expression using RT-qPCR assay. Nine selected genes (*ROC_gene110506*, *ROC_gene137099*, *ROC_gene151979*, *ROC_gene166081*, *ROC_gene19562*8, *ROC_gene206594*, *ROC_gene206941*, *ROC_gene246729*, and *ROC_gene253298*) were analyzed by RT-qPCR. Expression levels of target genes were calculated using the $2^{-\Delta\Delta Ct}$ method with the sugarcane serine/arginine repetitive matrix protein 1 coding gene (*SARMp1*) serving as an endogenous control. Values are presented as the mean ± standard deviation of three independent experiments with three technical triplicates each. RT-qPCR (left-axis) and RNA-seq (right-axis) data for the nine genes show a significant correlation ($p < 0.05$).

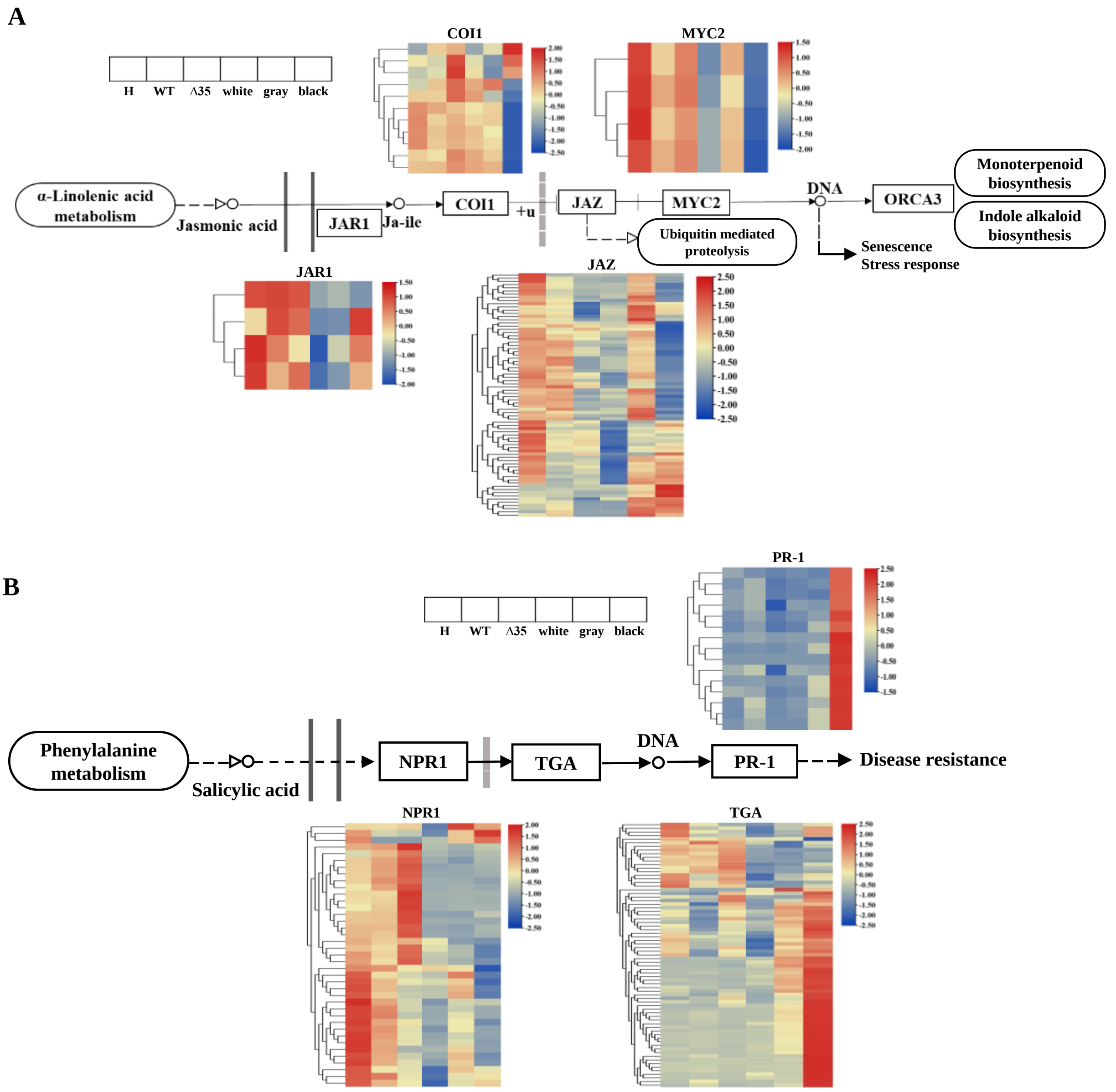

FIGURE S2 Transcriptional changes in the sugarcane DEGs associated with jasmonic acid and salicylic acid signal transduction. (A) Heatmap of genes involved in jasmonic acid signal transduction. Scale bar represents values in z-score TPM. The blue color scale (z-score) indicates lower expression, while the red color scale indicates higher expression. **(B)** Heatmap of genes involved in salicylic acid signal transduction.

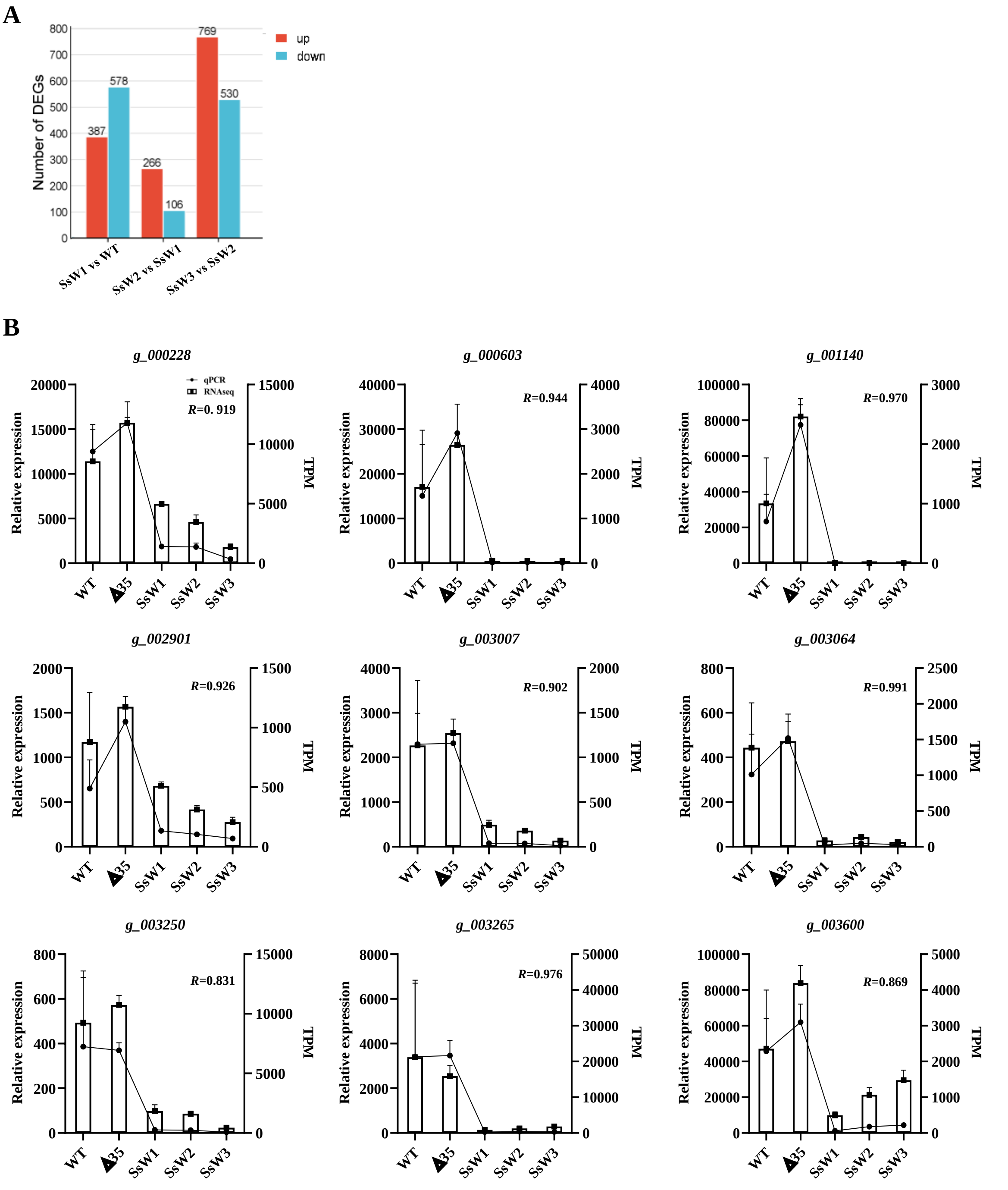

FIGURE S3 Differential gene expression of *S. scitamineum* across sorus segments and meristems infected by *RWTD1* mutants. **(A)** Number of differentially expressed genes in the RNA-seq analysis. **(B)** Validation of *S. scitamineum* gene expression using the RT-qPCR assay. Nine selected genes (*g_000628*, *g_000603*, *g_001140*, *g_002901*, *g_003007*, *g_003064*, *g_003250*, *g_003265*, and *g_003600*) were analyzed using the RT-qPCR assay. Expression levels of target genes were calculated using the $2^{-\Delta\Delta Ct}$ method, with the *S. scitamineum ACTIN* gene serving as an endogenous control. Values are presented as the mean ± standard deviation of three independent experiments with three technical triplicates each. RT-qPCR (left-axis) and RNA-seq (right-axis) data for the nine genes show a significant correlation ($p < 0.05$).
